# Dissociating Effects of Scrambling and Topicalization within the Left Frontal and Temporal Language Areas: An fMRI Study in Kaqchikel Maya

**DOI:** 10.3389/fpsyg.2017.00748

**Published:** 2017-05-09

**Authors:** Shinri Ohta, Masatoshi Koizumi, Kuniyoshi L. Sakai

**Affiliations:** ^1^Department of Basic Science, Graduate School of Arts and Sciences, The University of TokyoTokyo, Japan; ^2^Core Research for Evolutionary Science and Technology, Japan Science and Technology AgencyTokyo, Japan; ^3^Department of Linguistics, Graduate School of Arts and Letters, Tohoku UniversitySendai, Japan

**Keywords:** language, syntax, word order, scrambling, topicalization, inferior frontal gyrus, lateral premotor cortex, fMRI

## Abstract

Some natural languages grammatically allow different types of changing word orders, such as object scrambling and topicalization. Scrambling and topicalization are more related to syntax and semantics/phonology, respectively. Here we hypothesized that scrambling should activate the left frontal regions, while topicalization would affect the bilateral temporal regions. To examine such distinct effects in our functional magnetic resonance imaging study, we targeted the Kaqchikel Maya language, a Mayan language spoken in Guatemala. In Kaqchikel, the syntactically canonical word order is verb-object-subject (VOS), but at least three non-canonical word orders (i.e., SVO, VSO, and OVS) are also grammatically allowed. We used a sentence-picture matching task, in which the participants listened to a short Kaqchikel sentence and judged whether a picture matched the meaning of the sentence. The advantage of applying this experimental paradigm to an understudied language such as Kaqchikel is that it will allow us to validate the universality of linguistic computation in the brain. We found that the conditions with scrambled sentences [+scrambling] elicited significant activation in the left inferior frontal gyrus and lateral premotor cortex, both of which have been proposed as grammar centers, indicating the effects of syntactic loads. In contrast, the conditions without topicalization [-topicalization] resulted in significant activation in bilateral Heschl’s gyrus and superior temporal gyrus, demonstrating that the syntactic and phonological processes were clearly dissociated within the language areas. Moreover, the pre-supplementary motor area and left superior/middle temporal gyri were activated under relatively demanding conditions, suggesting their supportive roles in syntactic or semantic processing. To exclude any semantic/phonological effects of the object-subject word orders, we performed direct comparisons while making the factor of topicalization constant, and observed localized activations in the left inferior frontal gyrus and lateral premotor cortex. These results establish that the types of scrambling and topicalization have different impacts on the specified language areas. These findings further indicate that the functional roles of these left frontal and temporal regions involve *linguistic* aspects themselves, namely syntax versus semantics/phonology, rather than output/input aspects of speech processing.

## Introduction

There are natural languages that grammatically allow different types of changing word orders (Karimi, 2003). This phenomenon can be explained by *movement* of phrases, which is a key operation proposed in modern linguistics. A word order with the simplest syntactic structure is *syntactically canonical*, and word orders that are a result of a movement of phrases are non-canonical. The notion of such canonicity, as well as syntactic knowledge, is independent of the frequency/probability of usage, or learning of words (Chomsky, 1957). One type of movement is *object scrambling*, where an object (O) to be emphasized is extracted from the original position in a verb phrase and moved to a structurally higher position, skipping other phrases and resulting in more complex tree structures. In this article, we refer to “object scrambling” as simply “scrambling.” Scrambling is not allowed in English, but scrambled sentences are grammatical in Japanese. Although there are information structure distinctions (e.g., emphasis) related to scrambling, scrambling in Japanese does not change the grammatical relations (e.g., subject, direct object, and indirect object) and semantic roles (e.g., agent, patient, and experiencer) of a sentence (Fukui, 1993; Saito and Fukui, 1998).

Our previous study using functional magnetic resonance imaging (fMRI) revealed selective responses to scrambled sentences in the left frontal regions: the opercular and triangular parts (L. F3op/F3t) of the left inferior frontal gyrus (IFG), as well as the left lateral premotor cortex (L. LPMC) (Kinno et al., 2008). In our magnetoencephalography (MEG) study (Inubushi et al., 2012), we observed the effects of canonicity in the left IFG in response to more complex ditransitive sentences (i.e., those including a verb and two objects). We also demonstrated that the Degree of Merger (DoM) accounted for syntax-selective activations in the L. F3op/F3t (Ohta et al., 2013b). The DoM is the maximum depth of merged subtrees (i.e., Mergers) within an entire sentence, and it properly measures the complexity of tree structures. The DoM domain, i.e., the subtrees where the DoM is calculated, is an entire sentence when there is no constraint, but this changes dynamically in accord with syntactic operations and/or task requirements (Ohta et al., 2013a). Scrambling induces higher syntactic loads, because the DoM becomes at least one unit larger in accord with an additional branch for an extracted object, where the DoM domain also becomes larger covering entire sentences with a verb phrase (see Figure 6 of Ohta et al., 2013a). In addition to the L. F3op/F3t and L. LPMC, some fMRI and MEG studies have proposed that the left anterior temporal lobe (L. ATL) is also specialized in the construction of complex meaning (Poeppel et al., 2012), although effects of a movement of phrases have not been previously examined by those studies. By directly contrasting scrambled sentences with non-scrambled ones, the relative contribution of the L. F3op/F3t and L. ATL should be clarified.

Another type of movement is *topicalization*, in which, for example, a subject (S) or an object outside a verb phrase moves to a still structurally higher position to represent a topic, i.e., information that has already been mentioned in the discourse/context. In English, topicalization of an object generates a *non-canonical* word order (Radford, 2009), such as “*John read a book. That book, Mary read at school*,” in which a two-step movement of an object is involved. Here, the given information is presented as a topic at the initial position of the sentence, which makes sentence comprehension easier. Indeed, a sentence with the same movement of a non-topic noun phrase becomes ungrammatical: “^∗^*A book, Mary read at school*.” In the absence of topicalization, semantic/phonological loads and general auditory attention would become larger, because *all* words should be attended without prior information, rather than a particular topicalized word. Another possibility is that a topicalized sentence becomes semantically and phonologically marked, which may increase semantic/phonological loads in comparison with the canonical word order. By examining both effects of [±topicalization] in brain activation, we would be able to determine which of these effects is more prominent.

**FIGURE 1:**
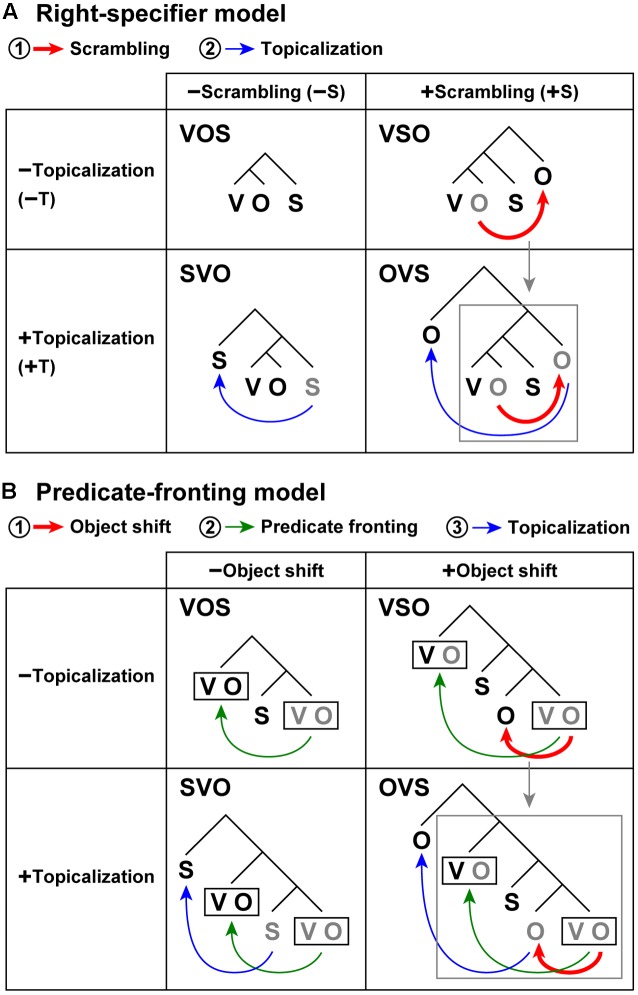
**Scrambling and topicalization induce *non-canonical* word orders. (A)** A right-specifier model assumes specifiers positioned at the right branches outside the verb phrase (Aissen, 1992; Koizumi et al., 2014). The VOS word order is syntactically canonical in Kaqchikel. Among the four possible word orders, the VSO and OVS word orders include scrambling [+scrambling], whereas VOS and SVO do not [-scrambling]. The SVO and OVS word orders include topicalization [+topicalization], and VOS and VSO do not [-topicalization]. The symbol ±S denotes the [±scrambling] condition, and ±T indicates the [±topicalization] condition, which are used in **Figures 3**, **4**. Scrambling (red arrow) and topicalization (blue arrow) are applied in sequential steps in this order. Scrambling induces higher syntactic loads, because the DoM becomes at least one unit larger in accord with an additional branch for an extracted object, where the DoM domain also becomes larger, covering the entire sentence with the verb phrase. On the other hand, topicalization does not produce additional syntactic loads, because the DoM remains always minimal, where the DoM domain is restricted to the peripheral structure of the topic and comment. After these *movements*, actual word orders (shown in black) are obtained. Gray letters denote the original positions of the phrases. **(B)** A predicate-fronting model. In this model, scrambling in the right-specifier model is replaced with the notion of *object shift*. The *predicate fronting* is assumed as a default and obligatory movement even for a canonical word order (Coon, 2010). A black rectangle denotes a predicate in each sentence. Object shift (red arrow), predicate fronting (green arrow), and topicalization (blue arrow) are applied in sequential steps in this order. For the OVS word order, both models propose that the object is further extracted while preserving the entire syntactic structures of the VSO word order, denoted by a gray arrow and a gray square.

While topicalization and scrambling are inseparable in rigid word-order languages such as English and Hebrew, they become separable in flexible word-order languages like Japanese. In the latter case, the DoM domain can be restricted to the peripheral structure of the topic and comment (i.e., the rest of the sentence), and thus topicalization does not produce additional syntactic loads, because the DoM remains minimal. An ERP study using topicalization and *wh*-questions in German reported that both constructions elicited a left-anterior negativity, which is typically interpreted as indexing an increase in memory burden (Felser et al., 2003). However, in German topicalized sentences, any effects due to a two- or multiple-step movement of an object should be considered, where the first-step of such a movement involves scrambling just as OVS in Kaqchikel (see **Figure 1A**). Moreover, topicalization may have enhanced memory burden, since no specific context was provided for each presented sentence in that study. Scrambling and topicalization are thus more related to syntax and semantics/phonology, respectively. According to psycholinguistic studies, the differences between these two types of movements do not seem to affect behavioral data (Sekerina, 2003). The use of fMRI would dissociate the effects of these movements among multiple language areas. Our previous studies have clarified that syntactic processing, i.e., movement or merger of phrases, activates the L. F3op/F3t (Kinno et al., 2008; Ohta et al., 2013a,b), while phonological loads and auditory attention activates the bilateral superior temporal gyrus (STG) (Suzuki and Sakai, 2003). Based on these studies, we hypothesized that the main effects of scrambling should activate the L. F3op/F3t and L. LPMC, while the main effects of topicalization would affect the bilateral temporal regions.

To dissociate the effects of [+scrambling] and [±topicalization], a flexible word-order language that grammatically allows four different word orders, i.e., [±scrambling, ±topicalization], should be targeted, which can be realized with Kaqchikel Maya (hereafter “Kaqchikel”), a Mayan language spoken in Guatemala. To our knowledge, the present study is the first to examine that [±scrambling] and [±topicalization] can be separated symmetrically within participants, sessions, and a language. In Kaqchikel, the syntactically canonical word order is verb-object-subject (VOS), but at least three non-canonical word orders (i.e., SVO, VSO, and OVS) are also grammatically allowed (**Figure 1**; García Matzar and Rodríguez Guaján, 1997; Brown et al., 2006). Previous neuroimaging and psycholinguistic studies have mainly targeted *SO languages*, where the S precedes the O in a canonical word order (e.g., SVO and SOV), such as English, Japanese, and German. Sentences with the non-canonical OS word order (e.g., scrambling) are more difficult to process than those with the canonical word order, while keeping all other factors such as semantic roles equal (Marantz, 2005). Indeed, fMRI studies have reported increased activation by non-canonical word orders in the left IFG (Bahlmann et al., 2007; Kinno et al., 2008; Kim et al., 2009), which may reflect the effects of scrambling. A neuroimaging study has described the enhanced neural effects of topicalization (Ben-Shachar et al., 2004), in which a two-step movement of an object is involved in Hebrew sentences as in English and German. Because no specific context was provided for each presented sentence in that latter study, it is also possible that topicalization artificially enhanced syntactic and semantic/phonological processes. Such an activation increase might be triggered by the OS word order itself, which is related to one of “irregular prominence factors of noun phrases,” such as Patient vs. Agent, Inanimate vs. Animate, etc. (Bornkessel et al., 2005; Grewe et al., 2006). To conclusively examine which of these accounts is correct in fMRI experiments, we have targeted the *OS language* of Kaqchikel, where the O precedes the S in a *canonical* word order. If the last possibility is correct, and the activation increase is triggered by the OS word order itself, then the canonical word order in Kaqchikel (VOS) would elicit higher activations than the non-canonical word order (VSO), which seems unlikely. We predict that VSO elicits higher activations than VOS.

Kaqchikel is a *head-marking language*, in which prefixes of a *verb* (i.e., a head in a sentence) specify numbers (singular or plural) and persons (first, second, or third) of the object/subject, whereas English, Japanese, and German are *dependent-marking languages*, in which noun phrases (dependents) that depend on a verb are always marked for subjects and objects when possible (like English pronouns). Regarding Mayan sentences, a right-specifier model has been proposed for the syntactic structures of a sentence (Aissen, 1992; Koizumi et al., 2014), assuming specifiers positioned at the right branches outside the verb phrase VO, in addition to specifiers of a complementizer phrase (e.g., “*that*”) positioned at the left branches (**Figure 1A**). For the canonical word order (VOS), the S is a specifier positioned at a right branch. Moreover, for the VSO and OVS word orders, scrambling of the O results in a right-specifier. On the other hand, for the SVO and OVS word orders, topicalization of the S or O results in a left-specifier of a complementizer phrase. These four word orders thus have the following factors: VOS [-scrambling, -topicalization], SVO [-scrambling, +topicalization], VSO [+scrambling, -topicalization], OVS [+scrambling, +topicalization]. Another linguistic study has proposed an alternative model, i.e., a predicate-fronting model (**Figure 1B**; Coon, 2010), which is basically consistent with the right-specifier model and will be discussed later.

**FIGURE 2:**
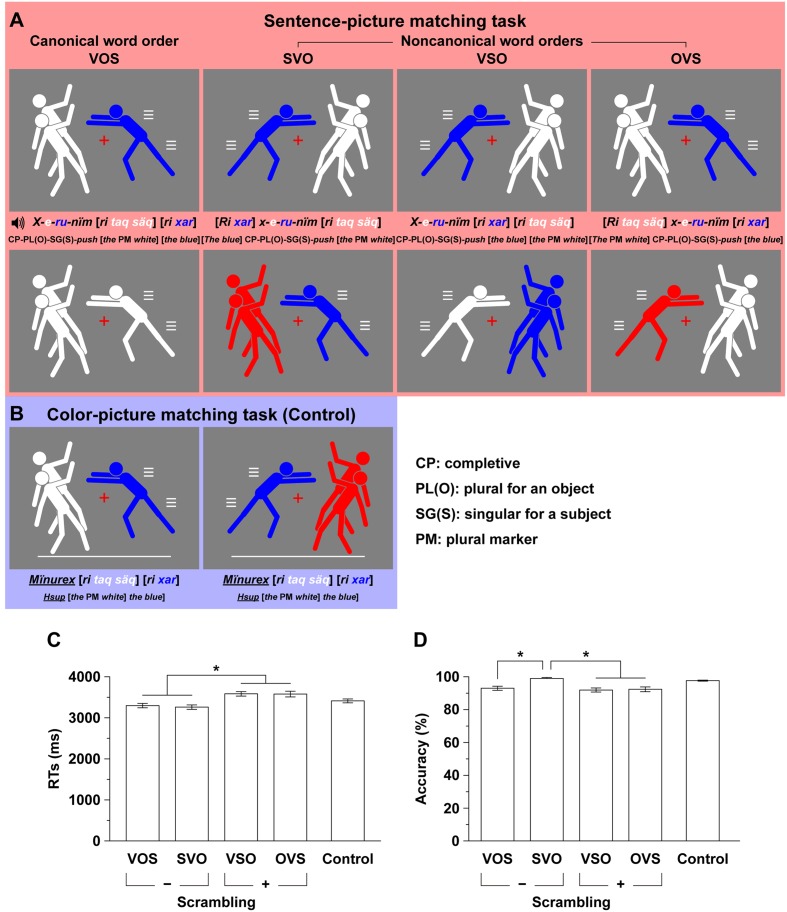
**An experimental paradigm with various grammatical word orders in Kaqchikel. (A)** A sentence-picture matching task (marked in red). We tested four task conditions based on the different word orders: VOS, SVO, VSO, and OVS; the VOS is the canonical word order, and the others are *non-canonical* word orders that are always grammatical. Each sentence with one of these word orders is auditorily presented, and a simultaneously presented picture consisted of a single man and two men with the same or different colors (white, blue, red, or black). The participants judged whether a picture matched the meaning of the sentence. For each example sentence in Kaqchikel and its word-by-word translation in English, a pair of matched and mismatched pictures are shown in the first and second rows, respectively. For display purposes, the blue and white words match the blue and white men in the pictures of the first row, respectively. **(B)** A color-picture matching task (the control task; marked in blue). Examples of matched and mismatched stimuli are shown in the left and right panels of the third row, respectively. The participants judged whether the colors in a picture matched the color words in the auditory stimuli, irrespective of their order. **(C)** Reaction times (RTs) from the onset of the picture for the sentence-picture and control tasks. Only correct trials were included. Error bars indicate the standard error of the mean (SEM) for the participants. ^∗^Corrected *p* < 0.05. **(D)** Accuracy for the sentence-picture and control tasks.

Based on our earlier investigations (Kinno et al., 2008, 2014), we used here a modified sentence-picture matching task, in which each participant listened to a Kaqchikel sentence and judged whether a picture matched the meaning of the sentence (**Figure 2**). The advantage of applying this experimental paradigm to an understudied language such as Kaqchikel is that it will allow us to validate the universality of linguistic computation in the brain.

## Basics of Kaqchikel Syntax

Kaqchikel is an *ergative language*, in which a subject of a transitive verb is marked by an *ergative case*, whereas an object of a transitive verb, as well as a subject of an intransitive verb, is marked by an *absolutive case*; here we used transitive verbs alone, with absolutive and ergative cases. The order of morphemes in a transitive verb is fixed as [Aspect-B-A-Verb stem] (Koizumi et al., 2014). In Kaqchikel syntax, ergative case markers are called set A, and absolutive case markers are called set B. As an “Aspect” prefix, we used a *completive* marker “*x-* (pronounced [∫])” alone (similar to a suffix *-ed* or *-en* as a perfect participle in English). Each set further makes agreement of number (singular and plural) and person (first, second, and third) between a verb and object/subject (i.e., absolutive/ergative). In the present study, we used only *third* persons with the following prefixes:

*ϕ-* (unmarked): singular for an absolutive case,
*e-*: plural for an absolutive case,
*r-*: singular for an ergative case, followed by a vowel-initial stem,
*ru-*: singular for an ergative case, followed by a consonant-initial stem,
*k-*: plural for an ergative case, followed by a vowel-initial stem, and
*ki-*: plural for an ergative case, followed by a consonant-initial stem.

## Materials and Methods

### Participants

We recruited 20 Kaqchikel speakers, who lived in Chimaltenango, Sololá, or Sacatepéquez (the *Departamentos* of Guatemala). They spoke the Northern, Western, or Southern Kaqchikel dialects spread in these regions (six, nine, or two participants for each dialect, respectively). Recruiting Kaqchikel speakers for the present fMRI experiment was challenging, because they were not accustomed to being the participants of experiments and felt fatigue due to the unfamiliar environment in Tokyo during their week-long stay. One participant retired from the experiment after the second run. Two participants whose accuracy under the OVS condition was <75% were excluded from the subsequent behavioral and fMRI analyses. We eventually analyzed 17 participants (7 males, 10 females; mean ± standard deviation [SD] age [years]: 32 ± 7.9), who correctly achieved >75% correct answers under each of the four sentence conditions. This criterion was based on a model-based clustering analysis (Fraley and Raftery, 2002), in which the classification into two clusters showed the highest likelihood.

All of the 17 participants were Kaqchikel-Spanish bilinguals (age of acquisition of Spanish: 4.9 ± 2.8 years), who showed right-handedness (laterality quotient: 72 ± 28) as determined by the Edinburgh Handedness Inventory (Oldfield, 1971). None had a history of neurological disease. There were three Kaqchikel speakers from Patzún (a town in Guatemala) in the present study. A linguistic study of Kaqchikel reported that speakers in Patzún prefer a subject-initial word order in transitives and intransitives (e.g., SVO, SV), and that they tend to interpret the VOS word order as an interrogative sentence (Clemens, 2013). However, interrogative sentences can be clearly distinguished by a rise in intonation (García Matzar and Rodríguez Guaján, 1997), and there was no interrogative sentence in our stimuli.

Thirteen participants acquired Kaqchikel from infancy, and the other four participants acquired Kaqchikel from the age of 5–8 years (age of acquisition of Kaqchikel: 2.4 ± 2.4 years). These four participants did not show any significant differences in the performance accuracy of the task compared to those who acquired Kaqchikel from infancy (two sample *t*-tests; [*t*(66) = 0.065, *p* = 0.95]). These four participants showed even shorter RTs [*t*(66) = 3.6, *p* = 0.0007], i.e., better performances. Moreover, a sub-analysis excluding these four participants showed basically similar activation patterns for the main effects of scrambling and topicalization.

During the experiments, translation was realized both ways through a Japanese-Spanish translator and a Spanish-Kaqchikel translator. To minimize the effects of Spanish usage during the experiment, we explained the stimuli and tasks to the participants in Kaqchikel through these translators. Prior to participation in the study, written informed consent was obtained from each participant after the nature and possible consequences of the study were explained. Approval for the experiments was obtained from the institutional review board of the University of Tokyo, Komaba Campus.

### Stimuli

For each trial of the sentence-picture matching task, auditory and visual stimuli were simultaneously presented. As auditory stimuli, a set of 64 original sentences was prepared for matched stimuli (16 sentences for each sentence condition), and a set of 64 sentences, consisting of 36 original sentences and 28 additional sentences (6–8 for each condition), was used for mismatched stimuli (16 sentences for each condition). Here we call the sentence-picture stimuli *mismatched*, when a picture does not match the meaning of a sentence. All sentences were grammatical, and word frequencies were controlled among the conditions.

Under each of the four sentence conditions with VOS, SVO, VSO, and OVS, we used the same set of verbs, nouns, a definite article “*ri*,” and a plural marker for nouns “*taq*.” We used only men with a definite article for nouns, but did not use an indefinite article or an animal, because a mixed use of definiteness (definite and indefinite) or animacy (human being, animal, etc.) of noun phrases may affect word orders (García Matzar and Rodríguez Guaján, 1997). Either a single man or two men in a sentence were represented by one of four colors: “*käq* (*red*), *q’ëq* (*black*), *säq* (*white*), and *xar* (*blue*)” in Kaqchikel (see **Figure 2A**). We used one of the following six Kaqchikel verbs: “*ch’äy* (*hit*), *jïk’* (*pull*), *nïm* (*push*), *oyoj* (*call*), *pixab’aj* (*bless*), and *xib’ij* (*surprise*).” A sentence example with VOS is “*X-e-ru-nïm* [*ri taq säq*] [*ri xar*] (*The blue pushed the whites*).”

In our stimuli, both the subject and object were humans. Note that the two sentences “*The blue pushed the white*” and “*The white pushed the blue*” (both men in singular or plural) cannot be distinguished by a prefix or noun phrase in Kaqchikel; the S and O cannot be formally determined. To resolve this type of ambiguity, each sentence included three men, which always consisted of one man (singular without “*taq*”) and two men (plural with “*taq*”).

All of the Kaqchikel sentences were spoken as a whole by a male native Kaqchikel speaker at a constant speed with natural prosody/intonation of declarative sentences, and those sentences were digitally recorded (16 bit; the normal audio cut-off, 44100 Hz). It should be noted that the spoken sentence contained rich information about prosody. With Sound Forge Pro 10 software (Sony Creative Software, Middleton, WI, USA), speech sounds were edited and their volumes were adjusted within the range from -50 to 0 dB full scale. A one-way repeated-measures analysis of variance (rANOVA) showed that the mean length of the auditory stimuli (2701 ± 168 ms) under each of the VOS, SVO, VSO, and OVS conditions was not significantly different [*F*(3,124) = 0.14, *p* = 0.94]. The input volume was set to a comfortable hearing level for each participant.

As visual stimuli, a set of 16 original pictures was prepared for matched stimuli, which were used for every sentence condition. For mismatched stimuli, 64 pictures were additionally made (16 pictures for each sentence condition), in which either or both of the color and number were changed from associated sentences. Half of the pictures depicted actions occurring from left to right, and the other half depicted actions occurring from right to left; colors of the single man and two men were also counterbalanced for both sides. The complexity of the pictures, as well as the frequency of action/color/number, was perfectly controlled among the sentence conditions.

All visual stimuli were presented against a gray background (**Figures 2A,B**). Each picture was presented for 5500 ms followed by a 500-ms blank interval. For fixation, a red cross was always shown at the center of the display, and the participants were instructed to keep their eyes on this position. Each auditory stimulus was presented 200 ms after the onset of each picture. The stimulus presentation and collection of behavioral data were controlled using the Presentation software package (Neurobehavioral Systems, Albany, CA, USA). The participants wore an MRI-compatible audio headset and an eyeglass-like MRI-compatible display (resolution, 800 × 600; VisuaStim Digital, Resonance Technology, Northridge, CA, USA).

### The Sentence-Picture Matching and Color-Picture Matching Tasks

In the sentence-picture matching task, each participant listened to a Kaqchikel sentence and judged whether a picture matched the meaning of the sentence (**Figure 2A**). To minimize the inclusion of short term memory, we presented the sentence while the participant looked at the picture. Trials with matched and mismatched stimuli were presented equally often (16 trials each for matched and mismatched stimuli under one condition). They responded by pressing one of two buttons that were aligned in a row (right for the matched pair and left for the mismatched pair) with their right thumb. Matching a picture with a sentence required the four following linguistic properties:

(1) color matching at the lowest lexical level,
(2) plurality matching with or without the plural marker “*taq*,”
(3) number and case (object/subject) matching, based on the verb prefixes, and
(4) sentence construction based on syntactic structures.

The first property involved lexico-semantics, and for the next two properties, checking syntactic/semantic features was essential. For the last property, syntactic decisions were required. The judgment of mismatch was possible either at the phrase presented second or at the phrase presented third of the heard sentence, with the same frequency. Note that the comparison between trials with the matched and mismatched stimuli was not within the scope of the present study.

In the sentence-picture matching task, mismatched stimuli (e.g., pictures in the middle row of **Figure 2A**) involved only one of the following four variations: (1) 24 pictures (four or eight under each condition) with one color alone, while two colors were used in the sentence (e.g., the leftmost picture); (2) 16 pictures (four under each condition) with a color different from that used in the sentence (e.g., the second and fourth pictures), thereby controlling the frequency of colors under each condition; (3) eight pictures (four under the VOS and VSO conditions), in which two colors were swapped between a single man and two men (e.g., the third picture); and (4) 16 pictures (eight each under the SVO and OVS conditions), in which the numbers of men were swapped. The first three variations of mismatched stimuli led to a violation in the linguistic properties mentioned above, thus requiring the comprehension of a whole sentence. The fourth variation, which involved attention to the exact verb prefixes due to the swapping of the number of men, may have required much higher loads than we had initially expected; we thus excluded those trials of mismatched stimuli from the subsequent behavioral and fMRI analyses.

In addition to the sentence-picture matching task, we also used a color-picture matching task (the *control* task), in which the participants judged whether colors in a picture matched the color words in the auditory stimuli (**Figure 2B**). By contrasting each of the four task conditions in the sentence-picture matching task with the control task at the first level of analysis, we could minimize the involvement of the first and second properties (see above) in any activation. For the auditory stimuli in the control task, we played the verb backward; as a result, the auditory stimuli contained the color words, plural marker, and definite articles. To indicate the control task, we added a white line at the bottom of the pictures, which were 128 different stimuli for the control task (64 each for matched and mismatched stimuli).

In the control task, mismatched stimuli involved only one of the following two variations: (1) 16 pictures with one color alone, while two colors were used in the auditory stimuli, and (2) 48 pictures with a color different from that in the auditory stimuli (e.g., the right picture of **Figure 2B**), thereby controlling the frequency of colors. General cognitive factors such as visual or auditory perception of the stimuli, matching, response selection, and motor responses were also controlled by the control task. We used the control condition as a baseline of the first-level analyses of the fMRI data to exclude these sensory and general cognitive factors as much as possible. The participants underwent short practice sessions before the task sessions to become fully familiarized with these tasks.

A single run of the task sessions (192 s) contained 16 “test events” of the sentence-picture matching task (four times each for the VOS, SVO, VSO, and OVS conditions), with inter-trial intervals of one control task. The order of the test events was pseudorandomized without repetition of the same condition, to prevent any condition-specific strategy. A single run contained 16 trials of the control task. Seven or eight runs were tested per one participant in a day. Only trials with participants’ correct responses were used for analyzing the RTs and fMRI data. For each participant, seven or eight runs without head movement were used for the behavior and fMRI analyses.

### MRI Data Acquisition

For the MRI data acquisition, the participant was in a supine position, and his or her head was immobilized inside the radio-frequency coil with straps. The MRI scans were conducted on a 3.0 T MRI system (GE Signa HDxt 3.0T; GE Healthcare, Milwaukee, WI, USA). We scanned 32 axial slices of 3-mm thick with a 0.3-mm gap, covering the volume range of -42.9 to 62.4 mm from the anterior to posterior commissure (AC-PC) line in the vertical direction, using a gradient-echo echo-planar imaging (EPI) sequence (repetition time [TR] = 2 s, echo time [TE] = 30 ms, flip angle [FA] = 90°, field of view [FOV] = 192 mm × 192 mm, resolution = 3 mm × 3 mm). In a single scan, we obtained 102 volumes where the first six images were discarded, which allowed for the rise of the MR signals.

After the completion of the fMRI session, high-resolution T1-weighted images of the whole brain (192 axial slices, 1.0 mm × 1.0 mm × 1.0 mm) were acquired from all participants with a three-dimensional fast spoiled gradient recalled acquisition in the steady state (3D FSPGR) sequence (TR = 8.6 ms, TE = 2.6 ms, FA = 25°, FOV = 256 mm × 256 mm). These structural images were used for normalizing the fMRI data.

### fMRI Data Analyses

The fMRI data were analyzed in a standard manner using SPM12 statistical parametric mapping software (Wellcome Trust Centre for Neuroimaging^1^) (Friston et al., 1995), implemented on MATLAB software (MathWorks, Natick, MA, USA). The acquisition timing of each slice was corrected using the middle slice (the 17th slice chronologically) as a reference for the EPI data. We realigned the time-series data in multiple runs to the first volume in all runs, and further realigned the data to the mean volume of all runs. The realigned data were resliced using seventh-degree B-spline interpolation, so that each voxel of each functional image matched that of the first volume. We removed runs that included data with a translation of >2 mm in any of the three directions and with a rotation of >1.4° around any of the three axes; these thresholds of head movement were empirically determined from our previous studies (Hashimoto and Sakai, 2002; Suzuki and Sakai, 2003; Kinno et al., 2008; Ohta et al., 2013b). For this reason, a single run was removed from one participant.

After alignment to the AC-PC line, each participant’s T1-weighted structural image was coregistered to the mean functional image generated during realignment. T1-weighted images were bias-corrected with light regularization, and segmented to the gray matter, white matter, cerebrospinal fluid, bone, other soft tissues, and air by using default tissue probability maps and the Segment tool in the SPM12, which uses an affine regularization to warp images to the International Consortium for Brain Mapping European brain template (Ashburner and Friston, 2005). Inter-subject registration was achieved with Diffeomorphic Anatomical Registration using the Exponentiated Lie algebra (DARTEL) toolbox in the SPM12 (Ashburner, 2007). The coregistered structural images were spatially normalized to the standard brain space as defined by the Montreal Neurological Institute (MNI) using DARTEL’s Normalize to MNI Space tool. All of the normalized structural images were visually inspected and compared with the standard brain for the absence of any further deformation. The realigned functional images were also spatially normalized to the MNI space by using DARTEL’s Normalize to MNI Space tool, which converted voxel sizes to 3 mm × 3 mm × 3 mm and smoothed the images with an isotropic Gaussian kernel of 9-mm full-width at half maximum.

In a first-level analysis (i.e., the fixed-effects analysis), each participant’s hemodynamic responses induced by the four sentence conditions as well as the control task for each session were modeled with a boxcar function with a duration of 5.5 s from the onset of each visual stimulus. The boxcar function was then convolved with a hemodynamic response function. Low-frequency noise was removed by high-pass filtering at 1/128 Hz. To minimize the effects of head movement, the six realignment parameters obtained from preprocessing were included as a nuisance factor in a general linear model. The images of the VOS - control, SVO - control, VSO - control, and OVS - control contrasts were then generated in the general linear model for each participant and used for the intersubject comparison in a second-level analysis (i.e., the random-effects analysis). To examine the activation of the regions in an unbiased manner, we adopted whole-brain analyses (Friston and Henson, 2006).

A repeated-measures analysis of covariance with *t*-tests was performed with two factors (scrambling × topicalization), the results of which were thresholded at uncorrected *p* < 0.0001 (*t* > 4.8) for the voxel level, and at corrected *p* < 0.05 for the cluster level, with topological false discovery rate (FDR) correction across the whole brain (Chumbley and Friston, 2009). We used the differences of accuracy between each sentence condition and control (e.g., VOS - control, SVO - control, VSO - control, and OVS - control) as a covariate of no interest (i.e., a nuisance factor). For the anatomical identification of activated regions, we basically used the Anatomical Automatic Labeling method^2^ (Tzourio-Mazoyer et al., 2002) and the labeled data as provided by Neuromorphometrics Inc.^3^ under academic subscription. For each region of interest, we extracted the mean percent signal changes for each participant from the local maxima (i.e., peak voxel) of each region in the second-level group analysis, using the MarsBaR-toolbox^4^.

## Results

### Behavioral Data

We used a two-by-two experimental design (factors: scrambling × topicalization). The behavioral data for the sentence-picture matching task are shown in **Figures 2C,D**. Under the sentence conditions, an rANOVA with these two factors on the RTs showed significant main effects of scrambling [*F*(1,16) = 153, *p* < 0.0001], but the main effect of topicalization and an interaction between these factors were not significant [topicalization, *F*(1,16) = 0.61, *p* = 0.45; interaction, *F*(1,16) = 0.24, *p* = 0.63]. Consistent with our theoretical predictions, these results indicated that the VSO and OVS conditions [+scrambling] produce greater syntactic loads than the VOS and SVO conditions [-scrambling].

Regarding the accuracy, the participants made reliable and consistent judgments, and the accuracy under every condition was higher than 90%. Under the sentence conditions, an rANOVA with these two factors on the accuracy showed significant main effects of scrambling and topicalization [scrambling, *F*(1,16) = 12, *p* = 0.0036; topicalization, *F*(1,16) = 9.5, *p* = 0.0072], and an interaction between these factors was marginally significant [*F*(1,16) = 4.4, *p* = 0.052]. *Post hoc* paired *t*-tests showed that the accuracy under the SVO condition was significantly higher than that under the other conditions (corrected *p* < 0.0024), indicating that SVO was the easiest condition.

### The Basic Design of the Functional Analyses

Here we outline the basic design of the main functional analyses. Based on the two-by-two experimental design (scrambling × topicalization), we first examined the main effects of scrambling [±S], i.e., (VSO + OVS) - (VOS + SVO), where the [*+*S] conditions mainly induced higher syntactic loads (see the Introduction). We then examined the main effects of topicalization [±T], i.e., (VOS + VSO) vs. (SVO + OVS), related to the semantic/phonological loads. To examine any effects associated with the accuracy for each condition, we also tested (VOS + VSO + OVS) - SVO, based on the behavioral results shown above.

To exclude any semantic/phonological effects of the object-subject word orders, we performed two direct comparisons while making the factor of topicalization constant: VSO [+S, -T] vs. VOS [-S, -T], and OVS [+S, +T] vs. SVO [-S, +T]. Lastly, we examined the activation profiles under the four sentence conditions in each of the identified regions of interest, and the results confirmed significant activation in these regions for a diagonal contrast of VSO [+S, -T] vs. SVO [-S, +T].

### The Cortical Activation Reflecting Syntactic Loads or Semantic/Phonological Loads

**FIGURE 3:**
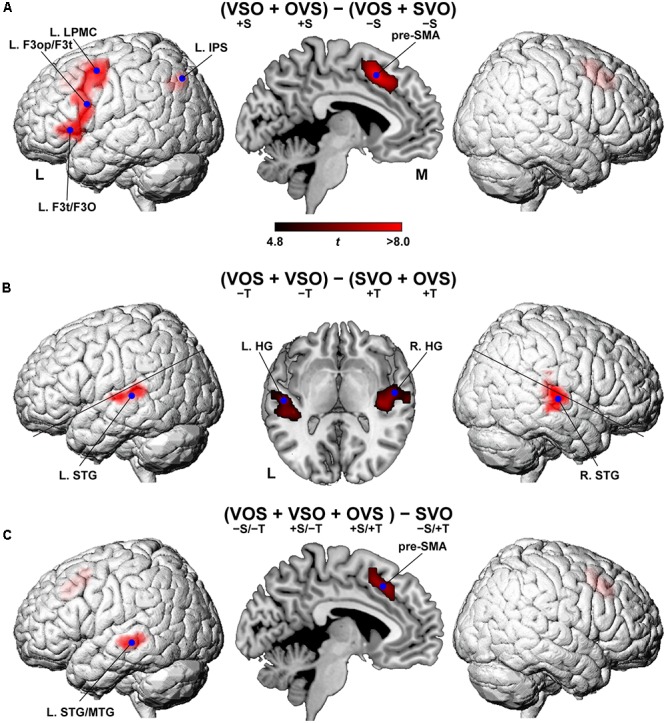
**Cortical activation modulated by the main effects of scrambling and topicalization. (A)** Regions identified by the main effects of scrambling, i.e., (VSO + OVS) - (VOS + SVO). Activations were projected onto the left (L) and right lateral surfaces of a standard brain (topological FDR-corrected *p* < 0.05). Medial sections are also shown. The activation of pre-SMA was also projected onto the lateral surfaces. Each blue dot indicates the local maximum of an activated region. See **Table 1** for the stereotactic coordinates of the activation foci. **(B)** Regions identified by the main effects of topicalization; i.e., (VOS + VSO) - (SVO + OVS). **(C)** Regions identified by the contrast of (VOS + VSO + OVS) – SVO.

The main effects of scrambling, i.e., (VSO + OVS) - (VOS + SVO), were observed in language areas such as the L. LPMC, L. F3op/F3t, and L. F3t/F3O (corrected *p* < 0.05) (**Figure 3A** and **Table 1**). Additional activation was observed in the pre-supplementary motor area (pre-SMA) and the left intraparietal sulcus (L. IPS). In contrast, the main effects of topicalization, i.e., (VOS + VSO) - (SVO + OVS), were observed in completely different regions: Heschl’s gyrus (HG) and the STG in both hemispheres (**Figure 3B** and **Table 1**). The reverse contrast, i.e., (SVO + OVS) - (VOS + VSO), did not show any significant activation (corrected *p* > 0.9). These results support the possibility that phonological loads and general auditory attention would become larger in the absence of topicalization (see the Introduction).

**Table 1:** Regions identified by the effects of word order.

Brain regions	BA	Side	*x*	*y*	*z*	*Z*	Voxels
**Main effects of scrambling: (VSO + OVS) - (VOS + SVO)**							
LPMC	6/9	L	-42	3	51	6.3	625
			-45	12	42	5.4	^∗^
			-36	12	33	4.9	^∗^
F3op/F3t	44/45	L	-48	18	27	5.1	^∗^
			-54	12	15	5.8	^∗^
F3t/F3O	45/47	L	-54	27	0	6.1	^∗^
pre-SMA	6/8/32	M	-6	15	48	6.5	352
			0	24	42	6.2	^∗^
IPS	7	L	-27	-69	42	5.2	71
**Main effects of topicalization: (VOS + VSO) - (SVO + OVS)**							
HG	41/42	L	-51	-21	6	5.6	213
STG	22	L	-51	-33	9	5.7	^∗^
			-63	-15	3	4.7	^∗^
HG	41/42	R	48	-15	6	5.6	326
			39	-24	6	5.8	^∗^
STG	22	R	66	-18	9	5.6	^∗^
			63	-18	-6	5.1	^∗^
**(VOS + VSO + OVS) - SVO**							
pre-SMA	6/8/32	M	-6	15	48	5.5	235
			0	27	42	5.7	^∗^
STG/MTG	22/21	L	-63	-21	-6	5.7	96
**VSO - VOS**							
LPMC	6/8	L	-36	6	57	4.5	112
			-48	0	45	5.4	^∗^
			-48	12	42	4.6	^∗^
F3op/F3t	44/45	L	-57	12	12	5.5	78
F3t/F3O	45/47	L	-54	24	0	4.4	^∗^
pre-SMA	6/8/32	M	-6	15	48	5.4	134
IPS	7/19	L	-12	-78	45	4.7	149
			-27	-69	39	5.5	^∗^
			-30	-75	24	4.7	^∗^
	7/19	R	18	-69	51	5.5	95
			30	-66	51	5.1	^∗^
**OVS - SVO**							
LPMC	6/8	L	-42	3	54	5.5	74
F3op/F3t	44/45	L	-51	18	27	4.7	35
F3t/F3O	45/47	L	-54	30	0	5.5	46
pre-SMA	6/8/32	M	-3	15	60	4.9	149
			-6	15	48	5.1	^∗^
			-3	27	42	5.5	^∗^

*Stereotactic coordinates (*x*, *y*, *z*) in the MNI space (mm) are shown for each activation peak of *Z* values. The threshold was set at corrected *p* < 0.05 for the cluster level. BA = Brodmann’s area; L = left; M = medial; R = right; LPMC = lateral premotor cortex; F3op/F3t/F3O = opercular/triangular/orbital parts of the inferior frontal gyrus; pre-SMA = pre-supplementary motor area; IPS = intraparietal sulcus; HG = Heschl’s gyrus; STG = superior temporal gyrus; MTG = middle temporal gyrus. The region with an asterisk is included within the same cluster shown one row above*.

In contrast, the contrast of (VOS + VSO + OVS) – SVO showed activation in the pre-SMA and left superior and middle temporal gyri (L. STG/MTG), sparing the lateral frontal regions (**Figure 3C** and **Table 1**). The pre-SMA activation replicated activation in the main effects of scrambling, while the L. STG/MTG activation was left-lateralized and located more ventrally than that in the main effects of topicalization.

**FIGURE 4:**
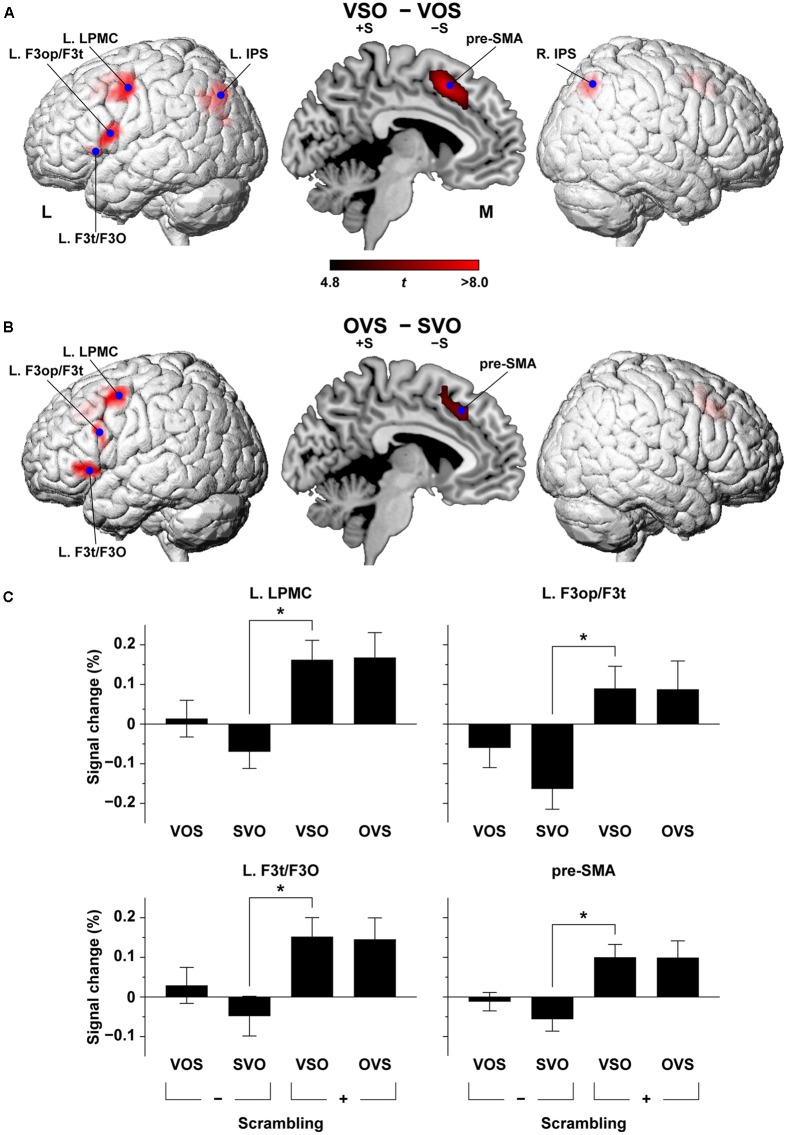
**Direct comparison of cortical activation between conditions**. The VSO - VOS contrast **(A)** and the OVS - SVO contrast **(B)**. Activations were projected onto the left (L) and right lateral surfaces of a standard brain (topological FDR-corrected *p* < 0.05). See **Table 1** for the stereotactic coordinates of the activation foci. **(C)** Histograms for the percent signal changes at the local maxima of the L. LPMC, L. F3op/F3t, L. F3t/F3O, and pre-SMA in OVS - SVO. The signal changes for VOS, SVO, VSO, and OVS are shown with reference to the baseline level of the control task. Error bars indicate SEM for the participants. ^∗^*p* < 0.0005.

We directly compared the cortical activation in VSO – VOS, and we observed localized activation in the L. LPMC, L. F3op/F3t, and L. F3t/F3O (**Figure 4A** and **Table 1**), i.e., the frontal language areas, which were consistent with the main effects of scrambling. Activation in the pre-SMA and L. IPS also replicated the main effects of scrambling, but the R. IPS was additionally activated. On the other hand, the reverse contrast, i.e., VOS - VSO, did not show any significant activation (corrected *p* > 0.9). In OVS - SVO, the overall activation pattern was similar to that in VSO – VOS (**Figure 4B** and **Table 1**). It is notable that the L. F3op/F3t activation shifted more dorsally (15 mm for the local maxima) in OVS – SVO. Compared with the main effects of scrambling, these activated regions were highly localized in such stringent contrasts as VSO - VOS and OVS - SVO.

At the local maxima of the L. LPMC, L. F3op/F3t, L. F3t/F3O, and pre-SMA in the second-level analysis, which were selected from the contrast OVS – SVO (shown as blue dots in **Figure 4B**), we examined the percent signal changes. In all of these regions, the overall activation profiles under the four sentence conditions were consistent. More specifically, the activations under VSO and OVS were always evident at the same level, whereas the activations under VOS and SVO were near or below the baseline level. Furthermore, the signal changes under VSO were significantly larger than those under SVO in each of the four regions (corrected *p* < 0.002).

The bilateral IPS activation, which was observed in VSO – VOS (shown as blue dots in **Figure 4A**), but not in OVS - SVO, may indicate the presence of an interaction. This effect was due to more activations under the VSO condition than the other conditions. A significant interaction was present in the R. IPS [*F*(1,16) = 6.5, *p* = 0.022], but not in the L. IPS [*F*(1,16) = 0.17, *p* = 0.69]. The VSO [+S, -T] condition reflected a synergistic effect of multiple linguistic factors, which may employ additional cortical regions like the bilateral IPS.

## Discussion

By using the sentence-picture matching task in the Kaqchikel language, we obtained four striking results. First, we found that the [+scrambling] conditions elicited significant activation in the left frontal regions of the L. LPMC, L. F3op/F3t, and L. F3t/F3O (**Figure 3A**), indicating the effects of syntactic loads in Kaqchikel, a head-marking and OS language. These results indicate that the L. LPMC, L. F3op/F3t, and L. F3t/F3O, but not the L. ATL, are crucial for a movement of phrases. Secondly, the [-topicalization] conditions resulted in significant activation in the bilateral HG and STG (**Figure 3B**), demonstrating that the syntactic and phonological processes were clearly dissociated within the language areas. Thirdly, the pre-SMA and L. STG/MTG were activated under the more demanding conditions other than SVO (**Figure 3C**), suggesting their supportive roles in syntactic or semantic processing. Fourthly, two direct comparisons of VSO – VOS and OVS – SVO showed *consistent* and localized activations in the L. LPMC, L. F3op/F3t, and L. F3t/F3O, as well as the pre-SMA (**Figures 4A,B**), while VOS – VSO did not show any significant activation. This last point fits the syntactic account for the selective activation in these frontal regions, excluding any semantic effects of the OS word order itself, which might be related to “irregular prominence factors of noun phrases” (see the Introduction). Our findings further indicate that the functional roles of these left frontal and temporal regions involve *linguistic* aspects themselves, namely syntax versus semantics/phonology, rather than output/input aspects of speech processing. Moreover, the present study with Kaqchikel clearly contributes to the concept that such universal operations as scrambling and topicalization are differentially processed in specified cortical regions.

“Merge” is a fundamental local structure-building operation proposed by modern linguistics (Chomsky, 1995), and is a key to syntactic processing. Neuroimaging studies have established that syntactic processing selectively activates the L. F3op/F3t and L. LPMC (Stromswold et al., 1996; Dapretto and Bookheimer, 1999; Embick et al., 2000; Hashimoto and Sakai, 2002; Friederici et al., 2003; Musso et al., 2003), indicating that these regions have a critical role as grammar centers (Sakai, 2005). Activations in the L. F3op/F3t and L. LPMC have also been observed in our studies using Japanese sentences with non-canonical word orders (Kinno et al., 2008). Moreover, our MEG studies showed a significant increase of responses in the L. IFG, which reflected predictive effects on a verb caused by a preceding object in a short sentence (Iijima et al., 2009; Inubushi et al., 2012; Iijima and Sakai, 2014). In the present study, we observed selective activation in the L. F3op/F3t and L. LPMC under the [+scrambling] conditions, which is consistent with these previous findings. Our results also support the explanation based on the DoM (Ohta et al., 2013a,b), in that the [+scrambling] conditions with the larger DoM enhanced the L. F3op/F3t and L. LPMC activations. It should be noted that activation in the L. LPMC, L. F3op/F3t, and L. F3t/F3O were more localized in both VSO – VOS and OVS – SVO, which excluded any differences in semantic/phonological loads. To our knowledge, our present findings are the first experimental evidence of linguistic computation that dissociates [+scrambling] and [-topicalization].

Here we observed activation in the bilateral HG and STG under the [-topicalization] conditions, which may reflect phonological loads and attention in the absence of topicalization. Our previous fMRI study revealed that the bilateral STG activations were selectively enhanced by phonological decision tasks (Suzuki and Sakai, 2003). The same study further demonstrated that the localized activations in the L. MTG were modulated by the presence of syntactic or semantic errors, which may enhance processing loads to correct sentences. Consistent with this possibility, here we observed the localized L. STG/MTG activation in the contrast of (VOS + VSO + OVS) – SVO.

In recent studies using a visual sentence-picture matching task similar to that used here, we tested 21 patients with a left frontal glioma and observed abnormal overactivity and/or underactivity in 14 syntax-related regions (Kinno et al., 2014, 2015). Those investigations also revealed three syntax-related networks: network I (syntax and its supportive system), network II (syntax and input/output interface), and network III (syntax and semantics). Functional and anatomical connectivity was observed within individual networks in normal controls, whereas in the agrammatic patients almost all of the functional connectivity exhibited chaotic changes. Moreover, the patients who showed normal performances showed normal connectivity between the L. F3op/F3t and L. IPS, as well as normal connectivity between the L. F3t and L. F3O, indicating that these pathways are the most crucial among the syntax-related networks. In the present study, we observed significant activation in the pre-SMA and L. IPS (**Figures 3**, **4**), which are included in network I (which consists of the L. F3op/F3t, pre-SMA, right lateral frontal regions, L. IPS, and right temporal regions). The consistent activation of the pre-SMA and L. IPS suggests their supportive roles in syntactic processing.

Another possible model for the syntactic structures of Mayan sentences has been proposed in a linguistic study: a predicate-fronting model (**Figure 1B**; Coon, 2010). In this model, which is more complex than the right-specifier model even for the canonical word order (VOS), *predicate fronting* is assumed as a default and obligatory movement. This model was based on similar syntactic analyses for another verb-initial language: Niuean, a Polynesian language that is markedly distant from Mayan languages (Massam, 2010). The notion of *object shift*, which precedes predicate fronting, replaces scrambling in the right-specifier model. Note that the verification of the right-specifier model or predicate fronting model was not within the scope of the present study; both models predict consistent results in our paradigm. The explanatory adequacy of these two models should be further examined in future experiments.

A previous fMRI study in Kaqchikel with a sentence plausibility judgment task has reported higher activation in the left IFG close to its border with the left middle frontal gyrus (BA 46) in the SVO - VOS contrast (Koizumi and Kim, 2016), clearly different activation from the present results. In that task, the participants listened to a sentence with a human (S) and an inanimate entity (O), and judged whether a sentence was semantically plausible or not, where no specific context was provided. The authors of that study interpreted this activation as the higher processing load related to more complex structures of SVO. They further argued that this higher load was related to the discourse-pragmatic requirements for the non-canonical SVO word order. A topicalized sentence incurs higher processing loads when presented out of context, as in the case of the sentence plausibility judgment task. In our present study, where both the subject and object were humans, we observed significant activation in the bilateral HG and STG as the main effects of topicalization, i.e., (VOS + VSO) - (SVO + OVS). This activation reflected increased phonological loads under the [-topicalization] conditions. Note that the reverse contrast did not show any significant activation, indicating that the [+topicalization] conditions had little syntactic effects where DoM remains minimal (see the Introduction). By naturally providing a discourse context as a picture, we were able to dissociate the main effects of topicalization related to semantics/phonology from those related to pragmatics.

In Kaqchikel, it has been reported that SVO is more frequently used than VOS (73% versus 15%) (Kubo et al., 2011). In that study, the native Kaqchikel speakers made a sentence describing a picture, which depicted a transitive action between a human agent and human/animal/inanimate patient. Although the concept of “basic word order” has been problematic (Brody, 1984), the word order with the simplest syntactic structure, i.e., syntactically canonical word order, is VOS (García Matzar and Rodríguez Guaján, 1997, p. 333). It has been suggested that “when examining the *basic* word order of Mayan languages, *syntactically* determined word order from the standpoint of syntactic complexity needs to be distinguished from *pragmatically* determined word order, commonly used for pragmatic purposes” (Yasunaga et al., 2015). This point is also related to our present observation that SVO was the easiest condition in our paradigm (**Figure 2D**). Both the higher production frequency and the higher accuracy of SVO may be caused by the effects associated with [+topicalization]. In a study using Japanese sentences, the production frequency of subject-topicalized sentences (S-*wa* OV) was several times higher than that of canonical sentences (S-*ga* OV), and the subject-topicalized sentences were more easily processed than the object-topicalized sentences (O-*wa* S-*ga* V) (Imamura and Koizumi, 2011); note that an S is considered as a default topic (Koster, 1978). These phenomena could be parallel to those for SVO versus canonical VOS in Kaqchikel, in that subject-topicalized sentences have lower semantic/phonological loads. On the other hand, one crucial difference between Japanese and Kaqchikel is that both canonical and subject-topicalized sentences are SOV in Japanese. Moreover, a string-vacuous movement, i.e., a movement without a change in the order of strings, is prohibited (Chomsky, 1986). Because both scrambling and topicalization are not string-vacuous movements in Kaqchikel, Kaqchikel is an ideal language for dissociating the effects of scrambling and topicalization. By targeting such understudied languages as Kaqchikel, we were able to integrate previous findings of neuroimaging and linguistic studies with our new findings, which will contribute to the understanding of the biological basis of language.

## Author Contributions

All authors listed, have made substantial, direct and intellectual contribution to the work, and approved it for publication.

## Conflict of Interest Statement

The authors declare that the research was conducted in the absence of any commercial or financial relationships that could be construed as a potential conflict of interest.

## Footnotes

1 http://www.fil.ion.ucl.ac.uk/spm/

2 http://www.gin.cnrs.fr/AAL2/

3 http://Neuromorphometrics.com/

4 http://marsbar.sourceforge.net/

## References

[B1] Aissen J. L. (1992). Topic and focus in Mayan. *Language* 68 43–80. 10.2307/416369

[B2] Ashburner J. (2007). A fast diffeomorphic image registration algorithm. *Neuroimage* 38 95–113. 10.1016/j.neuroimage.2007.07.007 17761438

[B3] Ashburner J. Friston K. J. (2005). Unified segmentation. *Neuroimage* 26 839–851. 10.1016/j.neuroimage.2005.02.018 15955494

[B4] Bahlmann J. Rodriguez-Fornells A. Rotte M. Münte T. F. (2007). An fMRI study of canonical and noncanonical word order in German. *Hum. Brain Mapp.* 28 940–949. 10.1002/hbm.20318 17274018 PMC6871458

[B5] Ben-Shachar M. Palti D. Grodzinsky Y. (2004). Neural correlates of syntactic movement: converging evidence from two fMRI experiments. *Neuroimage* 21 1320–1336. 10.1016/j.neuroimage.2003.11.027 15050558

[B6] Bornkessel I. Zysset S. Friederici A. D. Von Cramon D. Y. Schlesewsky M. (2005). Who did what to whom? The neural basis of argument hierarchies during language comprehension. *Neuroimage* 26 221–233. 10.1016/j.neuroimage.2005.01.032 15862222

[B7] Brody J. (1984). Some problems with the concept of basic word order. *Linguistics* 22 711–736. 10.1515/ling.1984.22.5.711

[B8] Brown R. M. Maxwell J. M. Little W. E. (2006). *La ütz awäch?: Introduction to Kaqchikel Maya Language.* Austin, TX: University of Texas Press.

[B9] Chomsky N. (1957). *Syntactic Structures.* The Hague: Mouton Publishers.

[B10] Chomsky N. (1986). *Barriers.* Cambridge, MA: The MIT Press.

[B11] Chomsky N. (1995). *The Minimalist Program.* Cambridge, MA: The MIT Press.

[B12] Chumbley J. R. Friston K. J. (2009). False discovery rate revisited: FDR and topological inference using Gaussian random fields. *Neuroimage* 44 62–70. 10.1016/j.neuroimage.2008.05.021 18603449

[B13] Clemens L. E. (2013). “Kaqchikel SVO: V2 in a V1 language,” in *Studies in Kaqchikel Grammar*, ed. Kenstowicz M. (Cambridge, MA: MITWPL), 1–23.

[B14] Coon J. (2010). VOS as predicate fronting in Chol. *Lingua* 120 354–378. 10.1016/j.lingua.2008.07.006

[B15] Dapretto M. Bookheimer S. Y. (1999). Form and content: dissociating syntax and semantics in sentence comprehension. *Neuron* 24 427–432. 10.1016/s0896-6273(00)80855-7 10571235

[B16] Embick D. Marantz A. Miyashita Y. O’neil W. Sakai K. L. (2000). A syntactic specialization for Broca’s area. *Proc. Natl. Acad. Sci. U.S.A.* 97 6150–6154. 10.1073/pnas.100098897 10811887 PMC18573

[B17] Felser C. Clahsen H. Münte T. F. (2003). Storage and integration in the processing of filler-gap dependencies: an ERP study of topicalization and *wh*-movement in German. *Brain Lang.* 87 345–354. 10.1016/S0093-934X(03)00135-4 14642537

[B18] Fraley C. Raftery A. E. (2002). Model-based clustering, discriminant analysis, and density estimation. *J. Am. Stat. Assoc.* 97 611–631. 10.1198/016214502760047131

[B19] Friederici A. D. Rüschemeyer S.-A. Hahne A. Fiebach C. J. (2003). The role of left inferior frontal and superior temporal cortex in sentence comprehension: localizing syntactic and semantic processes. *Cereb. Cortex* 13 170–177. 10.1093/cercor/13.2.170 12507948

[B20] Friston K. J. Henson R. N. (2006). Commentary on: divide and conquer; a defence of functional localisers. *Neuroimage* 30 1097–1099. 10.1016/j.neuroimage.2006.02.007 16635579

[B21] Friston K. J. Holmes A. P. Worsley K. J. Poline J.-P. Frith C. D. Frackowiak R. S. J. (1995). Statistical parametric maps in functional imaging: a general linear approach. *Hum. Brain Mapp.* 2 189–210. 10.1002/hbm.460020402

[B22] Fukui N. (1993). Parameter and optionality. *Linguist. Inq.* 24 399–420.

[B23] García Matzar L. P. Rodríguez Guaján P. B. J. O. (1997). *Rukemik ri Kaqchikel chi’: Gramática Kaqchikel [Kaqchikel Grammar].* Guatemala City: Cholsamaj.

[B24] Grewe T. Bornkessel I. Zysset S. Wiese R. Von Cramon D. Y. Schlesewsky M. (2006). Linguistic prominence and Broca’s area: the influence of animacy as a linearization principle. *Neuroimage* 32 1395–1402. 10.1016/j.neuroimage.2006.04.213 16769225

[B25] Hashimoto R. Sakai K. L. (2002). Specialization in the left prefrontal cortex for sentence comprehension. *Neuron* 35 589–597. 10.1016/s0896-6273(02)00788-2 12165479

[B26] Iijima K. Fukui N. Sakai K. L. (2009). The cortical dynamics in building syntactic structures of sentences: an MEG study in a minimal-pair paradigm. *Neuroimage* 44 1387–1396. 10.1016/j.neuroimage.2008.10.041 19041723

[B27] Iijima K. Sakai K. L. (2014). Subliminal enhancement of predictive effects during syntactic processing in the left inferior frontal gyrus: an MEG study. *Front. Syst. Neurosci.* 8:217 10.3389/fnsys.2014.00217 PMC4217366 25404899

[B28] Imamura S. Koizumi M. (2011). A centering analysis of word order in Japanese. *Tohoku Stud. Linguist.* 20 59–74.

[B29] Inubushi T. Iijima K. Koizumi M. Sakai K. L. (2012). Left inferior frontal activations depending on the canonicity determined by the argument structures of ditransitive sentences: an MEG study. *PLoS ONE* 7:e37192 10.1371/journal.pone.0037192 PMC3358340 22629366

[B30] Karimi S. (ed.) (2003). *Word Order and Scrambling.* Malden, MA: Blackwell Publishing.

[B31] Kim J. Koizumi M. Ikuta N. Fukumitsu Y. Kimura N. Iwata K. (2009). Scrambling effects on the processing of Japanese sentences: an fMRI study. *J. Neurolinguist.* 22 151–166. 10.1016/j.jneuroling.2008.07.005

[B32] Kinno R. Kawamura M. Shioda S. Sakai K. L. (2008). Neural correlates of noncanonical syntactic processing revealed by a picture-sentence matching task. *Hum. Brain Mapp.* 29 1015–1027. 10.1002/hbm.20441 17924553 PMC6871174

[B33] Kinno R. Ohta S. Muragaki Y. Maruyama T. Sakai K. L. (2014). Differential reorganization of three syntax-related networks induced by a left frontal glioma. *Brain* 137 1193–1212. 10.1093/brain/awu013 24519977

[B34] Kinno R. Ohta S. Muragaki Y. Maruyama T. Sakai K. L. (2015). Left frontal glioma induces functional connectivity changes in syntax-related networks. *Springerplus* 4:317 10.1186/s40064-015-1104-6 PMC4491091 26155456

[B35] Koizumi M. Kim J. (2016). Greater left inferior frontal activation for SVO than VOS during sentence comprehension in Kaqchikel. *Front. Psychol.* 7:1541 10.3389/fpsyg.2016.01541 PMC5064666 27790165

[B36] Koizumi M. Yasugi Y. Tamaoka K. Kiyama S. Kim J. Ajsivinac Sian J. E. (2014). On the (non)universality of the preference for subject-object word order in sentence comprehension: a sentence-processing study in Kaqchikel Maya. *Language* 90 722–736. 10.1353/lan.2014.0068

[B37] Koster J. (1978). “Why subject sentences don’t exist,” in *Recent Transformational Studies in European Languages*, ed. Keyser S. J. (Cambridge, MA: MIT Press), 53–64.

[B38] Kubo T. Ono H. Tanaka M. Koizumi M. Sakai H. (2011). VOS-gengo ni oite yuseisei ga gojun ni ataeru eikyo: kakuchikerugo ni okeru sengabyosha kadai de no kento [Animacy effects on word order in VOS language: evidence from a picture description task in Kaqchikel]. *IEICE Tech. Rep.* 111 19–24.

[B39] Marantz A. (2005). Generative linguistics within the cognitive neuroscience of language. *Linguist. Rev.* 22 429–445. 10.1515/tlir.2005.22.2-4.429

[B40] Massam D. (2010). V1 or V2?: on the left in Niuean. *Lingua* 120 284–302. 10.1016/j.lingua.2008.11.014

[B41] Musso M. Moro A. Glauche V. Rijntjes M. Reichenbach J. Büchel C. (2003). Broca’s area and the language instinct. *Nat. Neurosci.* 6 774–781. 10.1038/nn1077 12819784

[B42] Ohta S. Fukui N. Sakai K. L. (2013a). Computational principles of syntax in the regions specialized for language: integrating theoretical linguistics and functional neuroimaging. *Front. Behav. Neurosci.* 7:204 10.3389/fnbeh.2013.00204 PMC3866525 24385957

[B43] Ohta S. Fukui N. Sakai K. L. (2013b). Syntactic computation in the human brain: the Degree of Merger as a key factor. *PLoS ONE* 8:e56230 10.1371/journal.pone.0056230 PMC3577822 23437097

[B44] Oldfield R. C. (1971). The assessment and analysis of handedness: the Edinburgh inventory. *Neuropsychologia* 9 97–113. 10.1016/0028-3932(71)90067-4 5146491

[B45] Poeppel D. Emmorey K. Hickok G. Pylkkänen L. (2012). Towards a new neurobiology of language. *J. Neurosci.* 32 14125–14131. 10.1523/jneurosci.3244-12.2012 23055482 PMC3495005

[B46] Radford A. (2009). *Analysing English Sentences: A Minimalist Approach.* Cambridge: Cambridge University Press.

[B47] Saito M. Fukui N. (1998). Order in phrase structure and movement. *Linguist. Inq.* 29 439–474. 10.1162/002438998553815

[B48] Sakai K. L. (2005). Language acquisition and brain development. *Science* 310 815–819. 10.1126/science.1113530 16272114

[B49] Sekerina I. A. (2003). “Scrambling and processing: dependencies, complexity, and constraints,” in *Word Order and Scrambling*, ed. Karimi S. (Malden, MA: Blackwell Publishing), 301–324.

[B50] Stromswold K. Caplan D. Alpert N. Rauch S. (1996). Localization of syntactic comprehension by positron emission tomography. *Brain Lang.* 52 452–473. 10.1006/brln.1996.0024 8653390

[B51] Suzuki K. Sakai K. L. (2003). An event-related fMRI study of explicit syntactic processing of normal/anomalous sentences in contrast to implicit syntactic processing. *Cereb. Cortex* 13 517–526. 10.1093/cercor/13.5.517 12679298

[B52] Tzourio-Mazoyer N. Landeau B. Papathanassiou D. Crivello F. Etard O. Delcroix N. (2002). Automated anatomical labeling of activations in SPM using a macroscopic anatomical parcellation of the MNI MRI single-subject brain. *Neuroimage* 15 273–289. 10.1006/nimg.2001.0978 11771995

[B53] Yasunaga D. Yano M. Yasugi Y. Koizumi M. (2015). Is the subject-before-object preference universal? An event-related potential study in the Kaqchikel Mayan language. *Lang. Cogn. Neurosci.* 30 1209–1229. 10.1080/23273798.2015.1080372

